# The Edge-Disjoint Path Problem on Random Graphs by Message-Passing

**DOI:** 10.1371/journal.pone.0145222

**Published:** 2015-12-28

**Authors:** Fabrizio Altarelli, Alfredo Braunstein, Luca Dall’Asta, Caterina De Bacco, Silvio Franz

**Affiliations:** 1 DISAT and Center for Computational Sciences, Politecnico di Torino, Corso Duca degli Abruzzi 24, 10129 Torino, Italy; 2 Collegio Carlo Alberto, Via Real Collegio 30, 10024 Moncalieri, Italy; 3 Human Genetics Foundation, Via Nizza 52, 10126 Torino, Italy; 4 LPTMS, CNRS, Univ. Paris-Sud, Université Paris-Saclay, 91405 Orsay, France; Bangladesh University of Engineering and Technology, BANGLADESH

## Abstract

We present a message-passing algorithm to solve a series of edge-disjoint path problems on graphs based on the zero-temperature cavity equations. Edge-disjoint paths problems are important in the general context of routing, that can be defined by incorporating under a unique framework both traffic optimization and total path length minimization. The computation of the cavity equations can be performed efficiently by exploiting a mapping of a generalized edge-disjoint path problem on a star graph onto a weighted maximum matching problem. We perform extensive numerical simulations on random graphs of various types to test the performance both in terms of path length minimization and maximization of the number of accommodated paths. In addition, we test the performance on benchmark instances on various graphs by comparison with state-of-the-art algorithms and results found in the literature. Our message-passing algorithm always outperforms the others in terms of the number of accommodated paths when considering non trivial instances (otherwise it gives the same trivial results). Remarkably, the largest improvement in performance with respect to the other methods employed is found in the case of benchmarks with meshes, where the validity hypothesis behind message-passing is expected to worsen. In these cases, even though the exact message-passing equations do not converge, by introducing a reinforcement parameter to force convergence towards a sub optimal solution, we were able to always outperform the other algorithms with a peak of 27% performance improvement in terms of accommodated paths. On random graphs, we numerically observe two separated regimes: one in which all paths can be accommodated and one in which this is not possible. We also investigate the behavior of both the number of paths to be accommodated and their minimum total length.

## Introduction

The optimization of routing and connection requests is one of the main problems faced in traffic engineering and communication networks [1]. The need to deliver Quality of service (QoS) [2, 3] performances, when transmitting data over a network subject to overload and failures, requires both efficient traffic management and resource optimization.

Some aspects of these problems can be formalized using edge-disjoint path problems. For a given undirected graph and a set of sender-receiver communication requests among pairs of users, one is interested, in general terms, in finding paths between each pair, under the constraint that different paths cannot overlap on edges. Moreover, the additional requirement of minimization of the total path length can be considered. Apart from a purely theoretical interest [4], these problems find a wide range of applications: in very-large-scale-integration (VLSI) design, in admission control and virtual circuit routing and in all-optical networks. In VLSI design it is required to route wires on a circuit avoiding overlaps, along with minimizing the length of the wires [5, 6]. In admission control and virtual circuit routing [7–9] one needs to reserve in advance a given path for each communication request so that once the communication is established no interruption will occur. This has applications in real-time database servers, large-scale video servers [10–12], streaming data and bandwidth reservation in communication networks [13–16] and in parallel supercomputers. All these applications require high quality data transmission and full bandwidth exploitation. Routing via edge disjoint paths allows for an efficient bandwidth allocation among users because overlap avoidance means full bandwidth exploitation by each single user. An area that has attracted particular attention in the last decade is communication transmission in all-optical networks. Along an optical fiber different communications cannot be assigned the same wavelength to transmit data. Moreover, a unique wavelength must be assigned on all the edges contributing to the path assigned for a given communication. Routing communications under the above two requirements define the problem of routing and wavelength assignments (RWA) in this type of networks [17]. These two constraints suggest that a strategy that iteratively builds edge disjoint paths solutions could allow for a more efficient bandwidth management, namely by using an overall smaller number of wavelengths. This leaves available the remaining ones (according to the edge capacity) to be used either by new users entering the network or by allowing current users to exploit higher bandwidth. This strategy has indeed been applied using greedy [18] and genetic algorithms [19] with performances comparable to other methods based on integer linear programming, graph coloring or bin packing.

**Fig 1:**
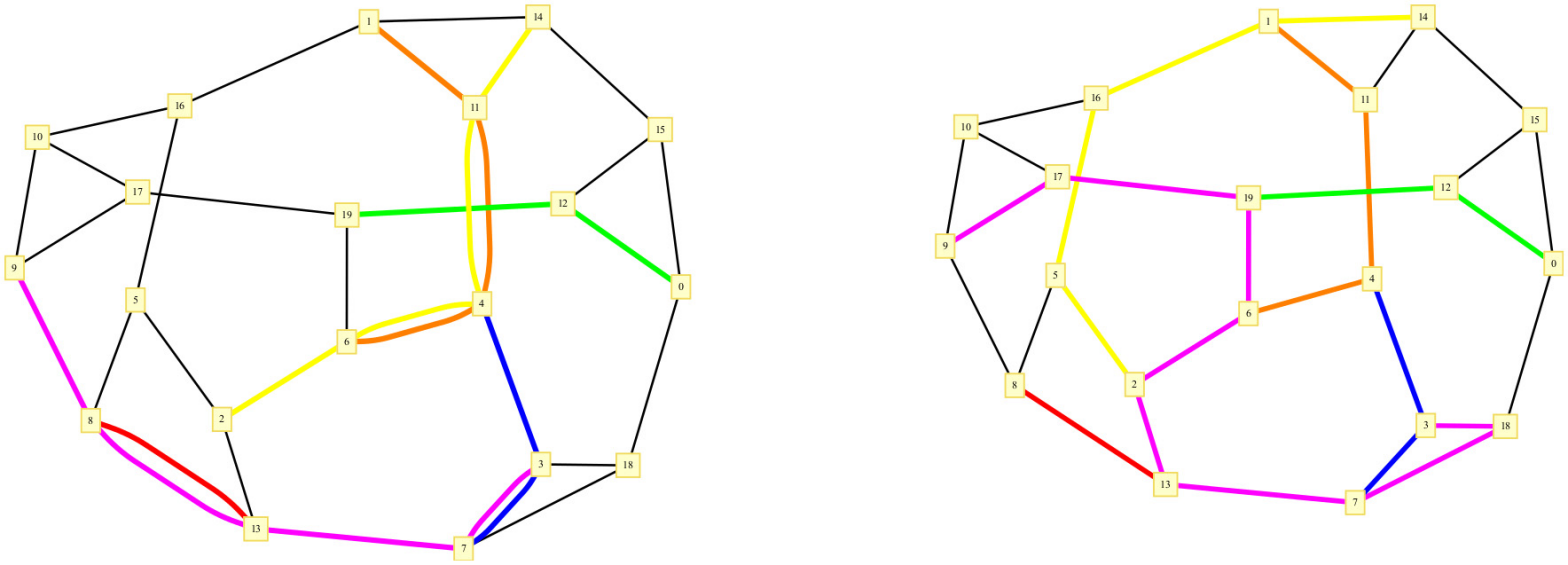
An instance of the MWEDP problem over a 3-regular random graph of *V* = 20 and *M* = 6: examples of solutions of the unconstrained (left) and optimal (right) MWEDP problem are displayed. In the latter, the purple communication is redirected along a longer path to avoid edge-overlap. The yellow one has two shortest paths of equal length (degeneracy) in the unconstrained case, but once the edge-disjointness is enforced this degeneracy is broken and only one of the two is optimal (right).

The problem that we will consider is the Minimum Weight Edge Disjoint Path (MWEDP) problem on undirected graphs. An instance of the MWEDP problem is defined by an (undirected) weighted graph G(V,E), with non-negative edge weights *w*, and by a set of *M* communication requests {(*S* ^*μ*^, *R* ^*μ*^)}_*μ* = 1, …, *M*_ between pairs of nodes. In Fig 1 we show an example of an instance of this problem on a regular random graph. The optimization problem consists in finding *M* pairwise edge-disjoint paths *π*_*μ*_, each one connecting a sender *S* ^*μ*^ with the corresponding receiver *R* ^*μ*^, while minimizing the total edge weight ∑_*μ*_ *w*(*π*_*μ*_), where *w*(*π*_*μ*_) = ∑_*e* ∈ *π*_*μ*__ *w*_*e*_. Note that the edge-disjointness of paths refers to undirected path edges, i.e. no two paths can share an edge, even if they go in different directions.

Two classical combinatorial edge-disjoint paths problems can be reduced to MWEDP. The classical (decisional) Edge-Disjoint Paths (EDP) problem consists in deciding if the *M* requests can be accommodated, disregarding path weights. The EDP problem is thus the one of deciding if a feasible solution of the MWEDP exits. The EDP problem is one of Karp’s original NP-complete combinatorial problems [20, 21]; and it has moreover been proved NP-complete in restricted conditions, as e.g. planar graphs and meshes. For a review, see e.g. [22, 23].

Some variants of the EDP problem are polynomial. First, the EDP problem is polynomial if the number *M* is constant, i.e., does not grow with *N* [4]. Second, if all *M* sender-receiver pairs are identical, it reduces to a maximum flow problem (Menger’s theorem, see e.g. [22]) and is thus also polynomial. A related problem is the (*S*, *R*) *M*-disjoint path problem, that is similar to an EDP problem but in which the specific sender-receiver associations are free. Specifically, here the input includes a set S⊂V (“senders”) and a set R⊂V (“receivers”) and one seeks *M* edge-disjoint paths connecting *S* and *R* (precisely, such that the set of all starting vertices is equal to *S*, and the set of all ending vertices is *R*). This problem is also polynomial as it can be trivially mapped into a problem with identical sender-receiver pairs: just add to the graph two new vertices (*s* ^⋆^ and *r* ^⋆^) and connect *s* ^⋆^ with all original senders in *S*, and *r* ^⋆^ with all original receivers in *R*. Now the (*S*, *R*) *M*-disjoint paths problem is equivalent to an EDP problem on the new graph with *M* identical sender-receiver pairs (*s*_1_, *r*_1_) = (*s*_2_, *r*_2_) = … = (*s*_*M*_, *r*_*M*_) = (*s* ^⋆^, *r* ^⋆^).

A second problem is Maximum Edge-Disjoint Paths (MDP), that consists in finding a maximum subset of the original *M* communications that can be accommodated, disregarding path weights, and can be reduced to the MWEDP problem by using zero edge weights for edges in the original graph and adding an extra set of *M* edges between pairs of sender and receiver with weight 1 (if the edge is already present, it suffices to add a new path of length two and total weight 1 instead of an edge). In undirected graphs, the MDP problem is NP-hard even on planar graphs [21].

Defining the approximation ratio of a given algorithm as the ratio between the result obtained in term of cost/profit by the algorithm and the optimal one (or viceversa depending on what order gives the maximum ratio), the MDP problem is hard to approximate in the worst case [23]; it has been proved that even an approximation with ratio $\Omega ({\left(\log n\right)}^{\frac{1}{2}-\varepsilon })$ (for *ϵ* > 0) is NP-hard [24], where *n* is the number of nodes in the graph. The best known approximation ratio for the number of accommodated paths is $O(\sqrt{n})$ [25]. The natural Linear Programming relaxation for the MDP problem has an $\Omega (\sqrt{n})$ integrality gap even on planar graphs [26]. Polylogaritmic approximation algorithms have been obtained for special classes of graphs, such as meshes and planar graphs [27–29]. Interestingly, for the MDP problem in two-dimensional meshes there is a randomized polynomial-time approximation algorithm that achieves a constant factor approximation with high probability [30].

Negative results on worst-case inapproximability did not stop progress on heuristic approaches. The problem has been studied intensely with a variety of classical techniques: heuristic greedy algorithms [13, 14, 18, 31], elaborated strategies using bin packing [32], integer/linear programming relaxations [33–36], post-optimization [37], Montecarlo local search [38], genetic algorithms [39–42], particle swarm optimization [43] and ant colony optimization [44], among them.

In this paper we propose a distributed algorithm to solve the MWEDP problem based on message-passing (MP) techniques (or cavity method) [45]. This method has been extensively employed to address problems in spin glass theory [46–48], combinatorial optimization [49] and more recently in routing problems on networks [50–53]. In the general case, the evaluation of the equations at the core of the MP technique requires, for each vertex *i* in the underlying graph, to solve a local combinatorial optimization problem on a star graph, performing a minimum over a set which is exponentially large in the number of neighbors of *i*. We propose here an efficient method to perform this calculation, by mapping it into a minimum-weight matching problem on a complete auxiliary graph with vertices in the set ∂*i* of neighbors of *i*, that can be solved by classical algorithms [54]. With this construction, each iteration of the MP equations can be computed in a time which is polynomial in the number of graph edges (and linear in average for sparse random graphs).

The MP algorithm is tested on computer-generated instances of different classes of random graphs to study the scaling properties with the system size and to compare the performances against a greedy algorithm. We also considered the EDP problem on some benchmark instances found in the literature, for which we could compare the message-passing results with those obtained using other types of algorithms: greedy, ant colony optimization [44] and Montecarlo local search [38].

The paper is organized as follows. In Section we define the EDP optimization problem, for which we present the message-passing equations in Section 1, together with the mapping on a matching problem that simplifies their actual implementation. Section 2 reports the results of simulations on random graphs and the scaling of the relevant quantities with the system size, while the comparison between the performances of the message-passing algorithm and other methods is discussed in Section 3. Conclusions are given in Section 4.

## Analysis

We introduce *M*-dimensional variables ${\bar{I}}_{ij}=\left(I_{ij}^{1},\cdots ,I_{ij}^{M}\right)$ with entries $I_{ij}^{\mu }\in \left\{\pm 1,0\right\}$ representing the communication passing along an edge:

$$\begin{array}{c} I_{ij}^{\mu }=\{\begin{array}{cc} 1, & \text{if}\;\text{communication}\;\mu \;\text{passes}\;\text{from}\;i\;\text{to}\;j,\\ -1, & \text{if}\;\text{communication}\;\mu \;\text{passes}\;\text{from}\;j\;\text{to}\;i,\\ 0, & \text{otherwise}. \end{array} \end{array}$$ (1)

Note that $I_{ij}^{\mu }=-I_{ji}^{\mu }$. We call these vectors *currents* as they must satisfy current conservation at each node *i* (Kirchhoff law):

$$\begin{array}{c} \sum_{j\in \partial i}I_{ij}^{\mu }+\Lambda_{i}^{\mu }=0,\;\forall \mu =1,\cdots ,M, \end{array}$$ (2)

where we defined for each node *i* and each communication *μ* a variable $\Lambda_{i}^{\mu }$ such that

$$\begin{array}{c} \Lambda_{i}^{\mu }=\{\begin{array}{cc} 1 & \text{if}\;i=S^{\mu }\text{,}\\ -1 & \text{if}\;i=R^{\mu }\text{,}\\ 0 & \text{otherwise}. \end{array} \end{array}$$ (3)

The constraint of edge-disjointness specifies that for each edge (*ij*), at most one of $I_{ij}^{\mu }$ is non-zero, therefore each vector ${\bar{I}}_{ij}$ can be parametrized by a variable taking 2*M* + 1 different values. Notice that the set of variables $\bar{I}={\left\{{\bar{I}}_{ij}\right\}}_{(ij)\in E}$ completely specifies the state of the network. In this multi-flow formalism, the MWEDP problem is a combinatorial optimization problem in which the global cost function $C\left(\bar{I}\right)=\sum_{(ij)\in E}f_{ij}\left(|\right|{\bar{I}}_{ij}\left||\right)$ depends additively on the total net current $\left|\right|{\bar{I}}_{ij}\left|\right|=\sum_{\mu }\left|I_{ij}^{\mu }\right|$ along the edges, and the edge-disjointness is ensured by defining

$$\begin{array}{c} f_{ij}\left(|\right|{\bar{I}}_{ij}\left||\right)=\{\begin{array}{cc} 0, & \text{if}\;\left|\right|{\bar{I}}_{ij}\left|\right|=0,\\ w_{ij}, & \text{if}\;\left|\right|{\bar{I}}_{ij}\left|\right|=1,\\ +\infty , & \text{if}\;\left|\right|{\bar{I}}_{ij}\left|\right|>1, \end{array} \end{array}$$ (4)

where $|I_{ij}^{\mu }|=0,1$ denotes the absolute value of $I_{ij}^{\mu }$ and here we consider symmetric weights *w*_*ij*_ = *w*_*ji*_. Thus configurations with more than one communication passing along an edge have infinite cost and, in the case of unit weights, the total cost $C(\bar{I})$, if finite, represents exactly the total path length, i.e. the number of edges traversed by paths. In order to allow communications to be absent, as mentioned in the mapping from EDP into MWEDP in the introduction, one can add an extra edge or path between sender and receiver. However, adding this extra path can in general worsen the approximation (e.g. even if the original graph was a tree, the modified one will normally be not). To avoid this issue, we will proceed as follows: to each sender or receiver *i* = *S* ^*μ*^, *R* ^*μ*^ we will add a new leaf *i*′ with the sole (i.e. without Eq (2)) constraint $I_{i^{\prime }i}^{\nu }=0$ if *ν* ≠ *μ* and with a sufficiently large cost $w_{i^{\prime }i}=\tilde{w}$. In this way, by paying cost $2\tilde{w}$ ($\tilde{w}$ for the sender and $\tilde{w}$ for the receiver), the communication can be always “accommodated” through these extra vertices without using the original graph edges.

## 1 The Message-Passing Algorithm

**Fig 2:**
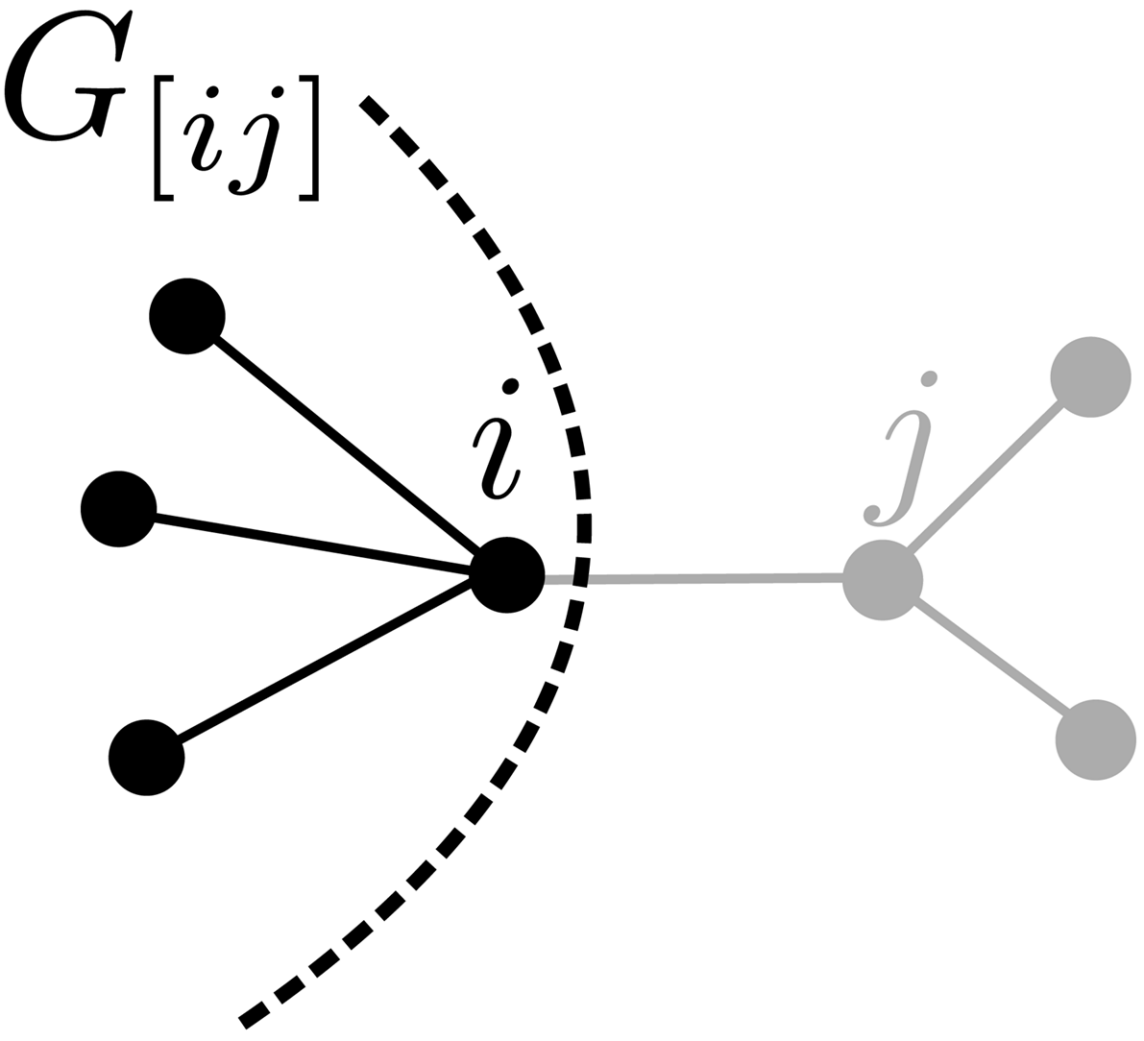
The modified cavity graph *G*_[*ij*]_.

On a tree, the model defined in Sec (that includes both the EDP and MWEDP problems) can be solved exactly by iteration using the following message-passing algorithm. Let us assume that *G* is a tree and consider the subtree *G*_[*ij*]_ defined by the connected component of *i* in *G*\(*ij*) (see Fig 2). On each *directed* edge (*ij*), we define $E_{ij}\left({\bar{I}}_{ij}\right)$ to be the minimum cost $C(\bar{I})$ among current configurations that satisfy Kirchoff’s laws on all vertices of *G*_[*ij*]_ given that we fix an input (or output) extra current ${\bar{I}}_{ij}$ entering (or exiting) node *i*. Because of the absence of cycles, it is possible to write a recursive equation for *E*_*ij*_ as a sum of cost contributions coming from neighbors of *i* in the subtree, plus the single cost contribution due to the current ${\bar{I}}_{ij}$ passing along edge (*ij*). We call these $E_{ij}\left({\bar{I}}_{ij}\right)$ quantities “messages” and they verify the *min-sum* recursion relation [47]:

$$\begin{array}{c} E_{ij}\left({\bar{I}}_{ij}\right)=\min_{\{{\bar{I}}_{ki}\}|constraint}\{\sum_{k\in \partial i\setminus j}E_{ki}\left({\bar{I}}_{ki}\right)\}+f_{ij}\left(|\right|{\bar{I}}_{ij}\left||\right) \end{array}$$ (5)

where *constraint* is the Kirchhoff law at node *i* and ∂*i* denotes the neighborhood of *i*. Note that in general $E_{ij}\left({\bar{I}}_{ij}\right)\neq E_{ji}\left({\bar{I}}_{ji}\right)$. Regarding the extra leaf *i*′ needed to allow communication *μ* on *i* = *S* ^*μ*^, *R* ^*μ*^ to be absent, we will simply define $E_{i^{\prime }i}\left({\bar{I}}_{i^{\prime }i}\right)=\infty$ if $I_{i^{\prime }i}^{\nu }>0$ for *ν* ≠ *μ* and $E_{i^{\prime }i}\left({\bar{I}}_{i^{\prime }i}\right)=f_{i^{\prime }i}\left(|\right|{\bar{I}}_{i^{\prime }i}\left||\right)$ otherwise. The opposite message *E*_*ii*′_ will be irrelevant as it doesn’t appear in the RHS of Eq (5).

The cavity method has been devised as an approximation on large random graphs to study the Boltzmann distribution, a probability measure defined by constraints and a given cost function [45, 47]. In random graphs, one sufficient condition for the validity of the cavity method depends on the decay of correlation between variables with their distance on the graph. However, the MP equations derived here (also called Max-Sum or Max-Product depending on some details of the formulation), are used to describe approximately optimal solutions rather than other characteristics of the probability space. For this limit, optimality can be proved even on general (i.e. non-random) graphs for certain simple (polynomially solvable) cases, including bipartite matching [55], minimum spanning tree [56] and maximum flow [57].

One can develop further this recursion to obtain a set of three types of message-passing equations, one for each type of node, i.e. for each value of $\Lambda_{i}^{\mu }$. A fixed point of these equations can be found by iteration from arbitrary initial values for the messages until convergence (see S1 Appendix for details about the convergence criterion used in the numerical implementation of the equations). Then, one can collect at each edge the incoming and the outgoing converged messages to find the optimal configuration ${\left\{{\bar{I}}_{ij}^{*}\right\}}_{(ij)\in E}$ such that:

$$\begin{array}{c} {\bar{I}}_{ij}^{*}{=}_{\bar{I}}\{E_{ij}\left(\bar{I}\right)+E_{ji}\left(-\bar{I}\right)-f_{ij}\left(|\right|\bar{I}\left||\right)\} \end{array}$$ (6)

where the last term is subtracted to avoid double counting of the cost of the single edge (*ij*).

### 1.1 The mapping into a weighted matching problem

The min-sum algorithm as in Eq (5) presents a computational bottleneck coming from the fact that for each output current ${\bar{I}}_{ij}$ there is a large number of possible neighborhood’s configurations ${\left\{{\bar{I}}_{ki}\right\}}_{k\in \partial i\setminus j}$ that are consistent both with the edge-disjoint constraints and with Kirchhoff’s law. In the calculation of the minimum in Eq (5) one needs in fact to consider all possible combinations of paths entering and exiting node *i*; the number of such combinations grows exponentially with the degree of node *i*. Nevertheless, the local optimization problem to consider is a somehow generalized edge-disjoint path problem in the neighborhood of *i* which is a star graph. As it happened for other disjoint-path problems on a star graph (see for instance [58]), it can be solved efficiently by reducing it to a maximum weight matching problem [54] on an auxiliary weighted complete graph $G_{i}^{\prime }$. The nodes of $G_{i}^{\prime }$ are the neighbors *k* ∈ ∂*i* and the (symmetric) weights matrix *Q* will be defined as

$$\begin{array}{c} Q_{kl}=-\min_{1\leq |\nu |\leq M}\{E_{ki}\left(\nu \right)+E_{li}\left(-\nu \right)\}+E_{ki}\left(0\right)+E_{li}\left(0\right) \end{array}$$ (7)

where $E_{kl}\left(\nu \right)=E_{kl}\left({\bar{I}}_{kl}\right)$ with $I_{kl}^{\mu }\equiv \delta_{\mu ,\nu }$ for *ν* > 0, $I_{kl}^{\mu }\equiv -\delta_{\mu ,\nu }$ for *ν* < 0 and $I_{kl}^{\mu }\equiv 0$ for *ν* = 0. Notice that this notation maps the *M*-dimensional vectors ${\bar{I}}_{ij}$ to the 2*M* + 1 possible current configurations *ν* allowed by the edge-disjointness constraint along a given edge. The computation of matrix *Q*, that requires *O*(*Mk* ^2^) operations, should be performed only once at the beginning of the update routine for node *i* ∈ *G*.

**Fig 3:**
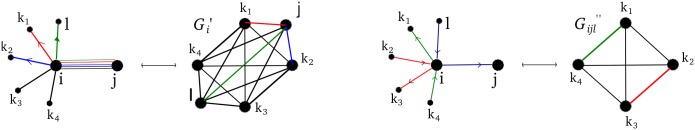
Mapping into a weighted matching problem. Left: intermediate step where $G_{i}^{\prime }$ is built. On the leftmost part we show an example of several communications passing along (*ij*) and exiting along the remaining neighbors *k* ∈ ∂*i* \ *j*. Right: the final step where $G_{ijl}^{\prime \prime }$ is built; the best configuration around node *i* when the blue current passes through (*ij*) is given by the minimum weighted matching on the complete auxiliary graph $G_{ijl}^{\prime \prime }$. Edges red and green represent the best matching, i.e. the configuration where two other communications enter/exit neighbors of *i* \ *j*.

Consider now a neighbor *j* ∈ ∂*i* and a given *μ* passing through edge (*ij*), we want to update $E_{ij}\left({\bar{I}}_{ij}\right)$. Assuming that current *μ* follows edge (*ji*) then (*il*) or viceversa, the optimal configuration for the current on the remaining neighboring edges is

$$\begin{array}{c} q_{jl}^{\min}=-M_{jl}+\sum_{k\in \partial i\setminus \{j,l\}}E_{ki}\left(0\right) \end{array}$$ (8)

where *M*_*jl*_ is the maximum weight of a matching on a complete graph $G_{ijl}^{\prime \prime }$ with *k* − 2 nodes, built from $G_{i}^{\prime }$ by removing nodes *j* and *l* (and all their incident edges). Recall that a matching is a subset of edges of $G_{ijl}^{\prime \prime }$ that do not share any vertex [54]. In this mapping, an edge (*kl*) of the matching corresponds to a current following the path (*k*, *i*, *l*) in any of the two directions (which of the two directions, and which of the communications, will be determined by the argmax of Eq (7)). The constraint that no two communications employ the same edge translates directly into the matching constraint. Hence, the computation of the update rule for the MP equations of the edge disjoint path problem translates into the solution of a standard (polynomial) combinatorial optimization problem, i.e. maximum weight matching. In Fig 3 we give a diagrammatic representation of the mapping. Note that in the maximum matching problem, edges in the input graph with negative weight can be simply removed. Notice that the neighboring current *ν* can also be a priori equal to *μ* in this algorithm, because the configurations where *μ* appears in more than one pair of edges will be eliminated in the minimization calculation as they have higher cost in our formulation. The minimum weight is thus independent of *μ*, i.e. of which message we are updating, a fact that allows reducing the complexity of the algorithm by a factor *M*.

Finally one needs to minimize over *l* given the matrix *q* ^min^:

$$\begin{array}{c} E_{ij}^{t+1}\left(\mu \right)=\min_{l\;\in \;\partial i\;\backslash j}\{E_{li}^{t}\left(\mu \right)+q_{jl}^{\min}\}+c_{ij}\left(\mu \right) \end{array}$$ (9)

where *c*_*ij*_(*μ*) is the cost of edge (*ij*), that in our case is 0 if *μ* = 0 and *w*_*ij*_ otherwise. We can notice that in order to evaluate each term inside the brackets we need to perform a matching optimization on each of the (*k* − 2)-node complete graphs $G_{ijl}^{\prime \prime }$ built ∀*l* ∈ ∂*i*∖*j*. Each of these matching routine has complexity *O*(*k* ^3^ log *k*) [59] and there are *O*(*k* ^2^) possible combinations of *j* and *l*. Reminding that we first need to evaluate the weight matrix *Q*, the overall complexity of this algorithm will be:

$$\begin{array}{c} O(k^{5}\log k+Mk^{2}) \end{array}$$

which is polynomial in the variables *k* and *M*. Once we have performed this whole procedure, we get all the information we need to calculate the 2*M* + 1 components of the update messages $E_{ij}^{t+1}\left(\mu \right)$, for each *j* ∈ ∂*i*, adding a term *O*(*kM*) to the final complexity (which is nonetheless negligible compared to the previous two).

The case of *μ* = 0, in which no current passes through edge (*ij*) regardless of what happens on the other edges, is addressed by calculating a matching on the (*k* − 1)-node complete graph composed of all nodes *l* ∈ ∂*i*∖*j*. If *i* is either a sender or a receiver, i.e. $\Lambda_{i}^{\mu }\in \left\{\pm 1\right\}$ for a given *μ* ∈ [1, …, *M*], the same computation can be performed provided that an auxiliary node, indexed by the communication label *μ* is added to the original graph *G* and connected to node *i*, such that its exiting messages will be fixed once at the beginning in the following way and never updated: *E*_*μi*_(*ν*) = −∞ if 0 < *ν* = *μ* (sender) or 0 < −*ν* = *μ* (receiver), and *E*_*μi*_(*ν*) = +∞ otherwise.

### 1.2 The role of reinforcement

**Fig 4:**
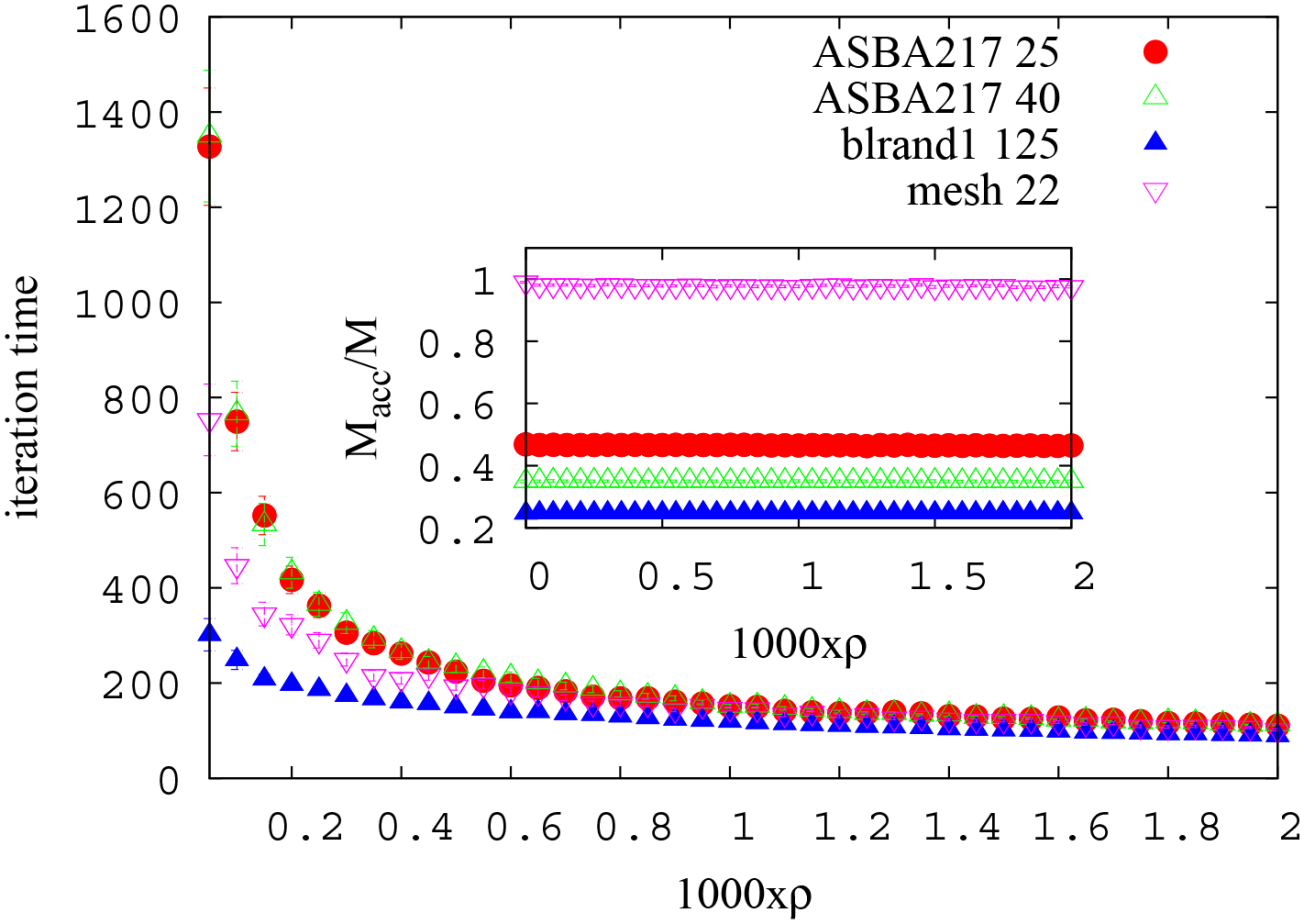
Reinforcement performance. Number of iterations to reach convergence as a function of reinforcement parameter *ρ* on BRITE graphs AS-BA217 (V = 100) with *M* = 25,40, blrand1 (V = 500) with *M* = 125 and mesh 15x15 with *M* = 22. Inset: the number of accommodated paths *M*_*acc*_ is substantially unchanged in the range of parameter values under study.

In order to aid and speed-up convergence of the MP equations, we used a reinforcement technique [60, 61], in which a set of external local fields

$$\begin{array}{c} h_{ij}^{t}\left(\mu \right)=E_{ij}^{t}\left(\mu \right)+E_{ji}^{t}\left(-\mu \right)-c_{ij}\left(\mu \right) \end{array}$$ (10)

act on the messages gradually biasing them to align with themselves. The reinforcement is introduced by promoting edge costs to become communication-dependent quantities defined as linear combinations of the cost at the previous time-step and the reinforcement local fields:

$$\begin{array}{c} c_{ij}^{t+1}\left(\mu \right)=c_{ij}^{t}\left(\mu \right)+\gamma_{t}h_{ij}^{t}\left(\mu \right) \end{array}$$ (11)

with $c_{ij}^{0}\left(\mu \right)=c_{ij}\left(\mu \right)$. This cost will then be inserted into equation Eqs (9) and (10) to replace the term *c*_*ij*_(*μ*). This has the effect to lead the messages to converge faster, gradually bootstrapping the system into a simpler one with large external fields. In practice we choose *γ*_*t*_ = *tρ* and one has to choose the growth rate of *γ* by tuning the reinforcement parameter *ρ*, that controls the trade-off between having a faster convergence and reaching a better solution. We tested *ρ* on instances on three types of graphs to finally choose to fix it to *ρ* = 0.002 in the rest of the simulations. In Fig 4 we could notice that this value achieves comparable results (inset) in terms of *M*_*acc*_/*M* to lower *ρ* in less time.

**Fig 5:**
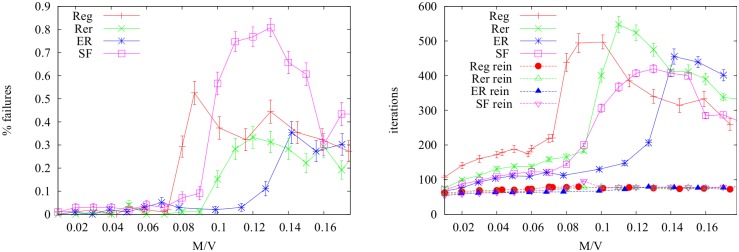
Left: Fraction of instances in which convergence standard MP fails (reinforced MP always converged in our experiments). Right: number of iterations for convergence for standard MP and reinforced MP (*ρ* = 0.002) in case of random graphs of *V* = 1000 and 〈*k*〉 = 3 as a function of *M*/*V*. Notice how the reinforcement term, besides ensuring convergence, greatly improves the convergence time.

In Fig 5(left) we report the number of converged instances (over 100 realizations) for standard MP (without reinforcement) on four types of random graphs (as described in the next section) and fixed size *V* = 1000 and average degree 〈*k*〉 = 3. The convergence failure of the standard MP increases considerably with *M*/*V* until it reaches a peak value, then it decreases. On the contrary, when reinforcement is used, convergence is always achieved in less than 100 steps (right panel).

## 2 Results on random graphs

First, we tested the MP algorithm on various types of random graphs, with fixed size V=|V|=1000 and average degree 〈*k*〉 = 3,5,7: regular random graphs (Reg), Erdős-Rényi random graphs (ER) [62], random graphs with power-law distribution (SF) [63] and a set of graphs (RER) obtained adding edges independently with probability *p* starting from a *k*_0_-regular random graph (for large *V*, the final average degree of such graphs is 〈*k*〉 = *k*_0_ + *d*, with *d* = *pV*). We compared the performance with a multi-start greedy algorithm (MSG) [44]. This heuristic algorithm calculates paths by iteratively choosing a (random) communication *μ*, finding the corresponding shortest path and removing the edges belonging to the path from the graph. The process is repeated until either there are no paths left to be routed or no communications can be accommodated anymore in the graph. The multi-start version repeats the same procedure a given number of times and keeps the best solution in terms of *M*_*acc*_, the number of accommodated paths. A bounded-length version [64] of MSG has been used to develop an iterative algorithm to solve the RWA using EDP in [18]: its performance was comparable to the one obtained using a linear programming solver on graphs of small sizes (*V* ≤ 40) but with faster execution times.

**Fig 6:**
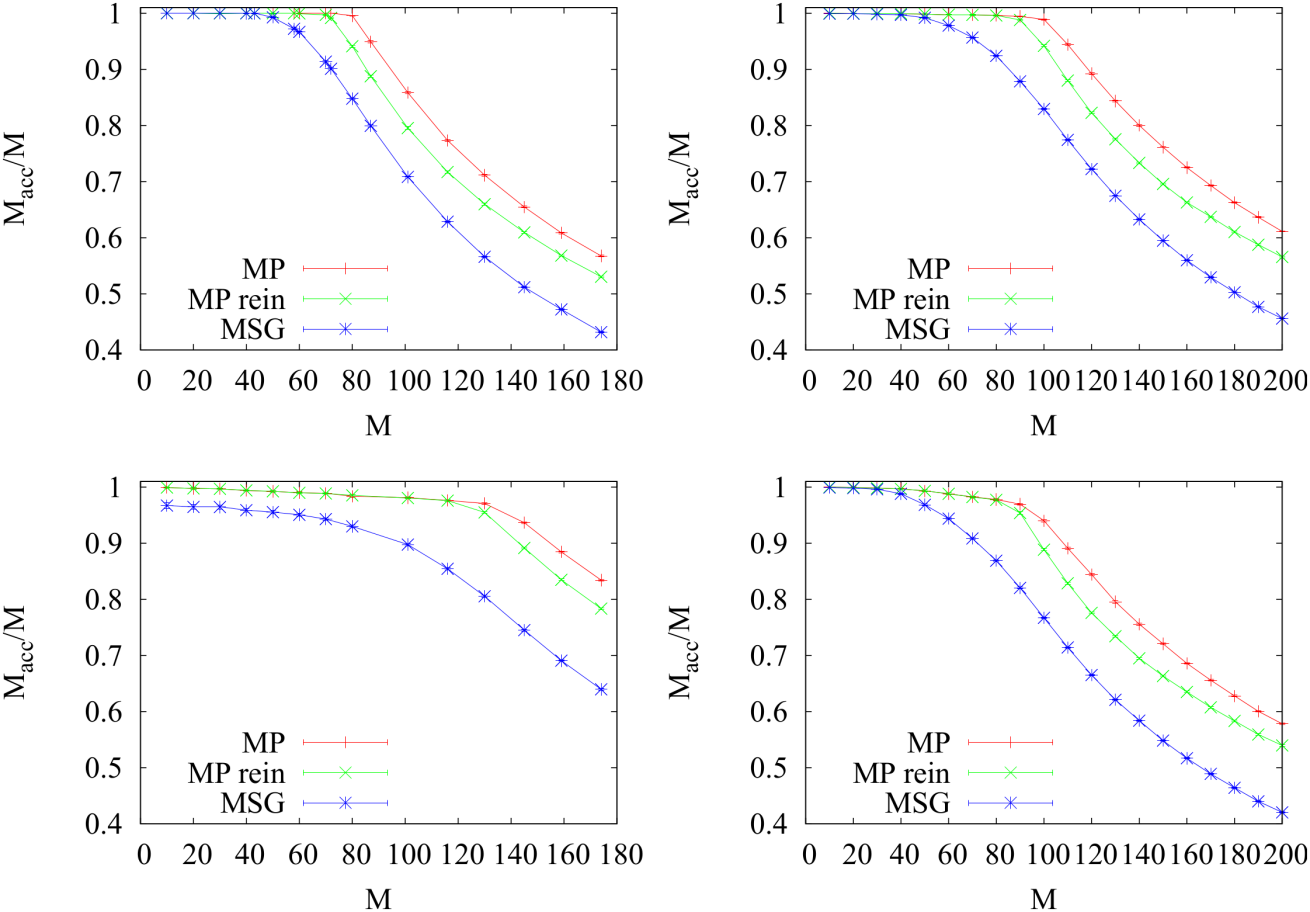
MP vs greedy performance. We plot the performance in terms of *M*_*acc*_/*M* for (from top to bottom) regular, RER, ER and SF graphs of fixed size *V* = 10^3^ and average degree 〈*k*〉 = 3. Error bars are smaller than the size of the symbols. A fast reinforcement parameter *ρ* = 0.002 for MP reinf was used.

**Fig 7:**
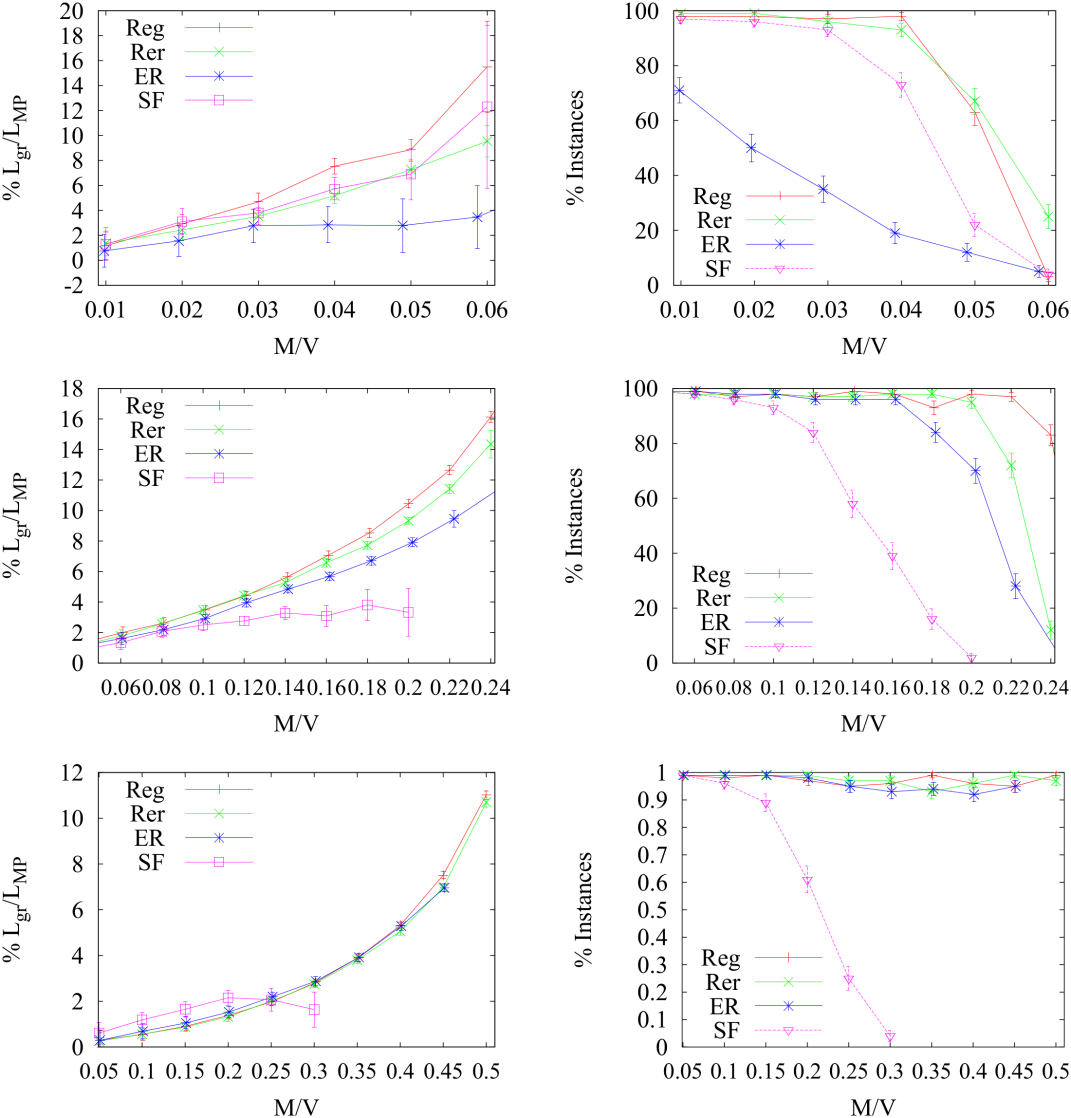
Length performance. We plot (left) the relative performance of MSG over MP in terms of total length of the solution paths: *y* = 100(*L*_*g*_/*L*_*MP*_ − 1). Here *L*_*g*_ and *L*_*MP*_ denote the total path lengths calculated with MSG and MP respectively. We use Reg, RER, ER and SF graphs of fixed size *V* = 10^3^ and average degree 〈*k*〉 = 3,5,7 (from top to bottom). On the right we report the number of instances where the two algorithms find the same solution in term of *M*_*acc*_/*M* over 100 realizations.

**Fig 8:**
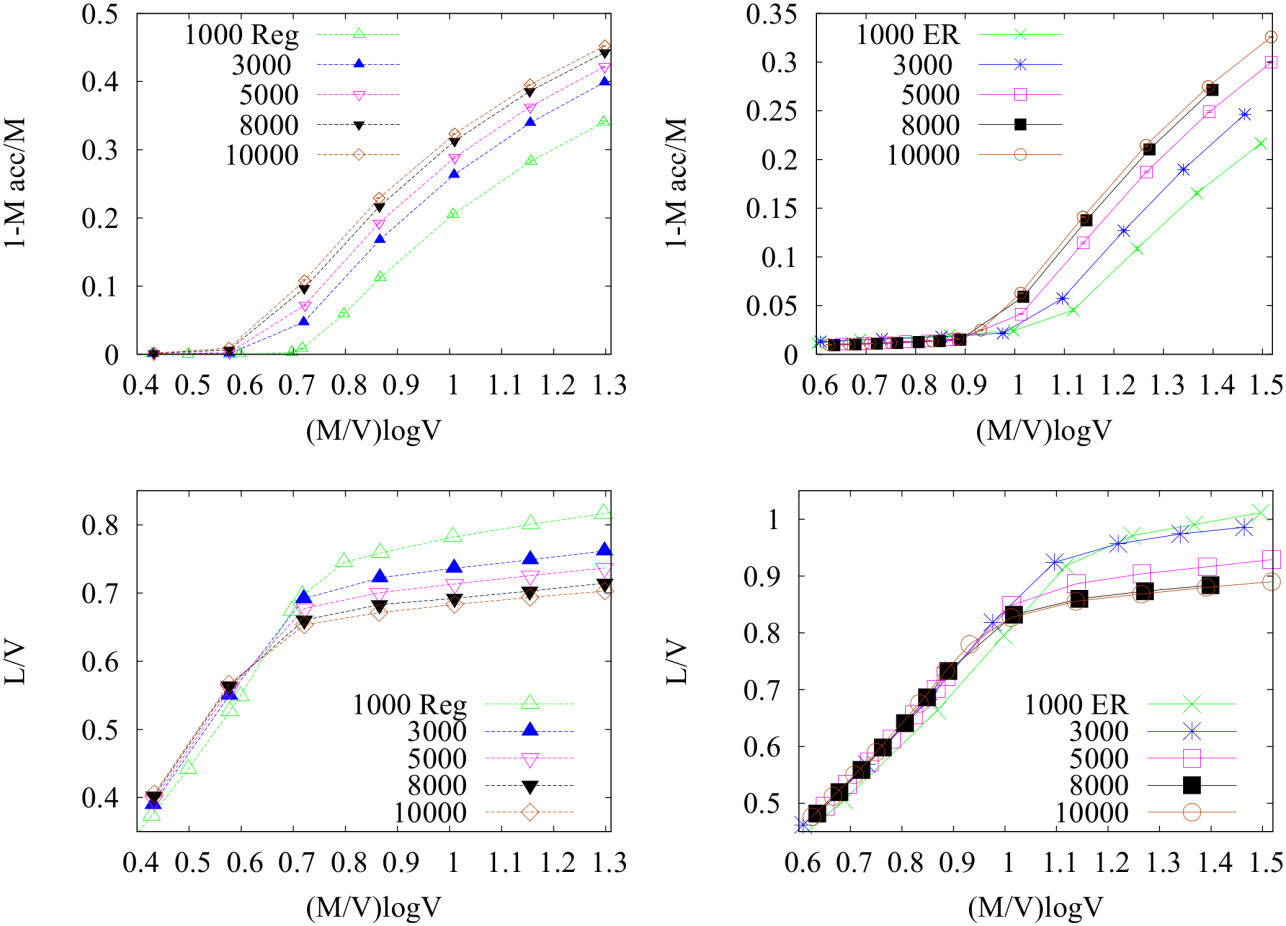
Finite-size effects. We plot 1 − *M*_*acc*_/*M* (top) and the total length per node *L*/*V* (bottom) for Reg (left) snd ER (right) graphs as a function of the scaling variable $\frac{M\log V}{V}$. We can notice the finite-size effects decreasing with system size leading to the curves corresponding to the biggest graphs *V* = 8000,10000 to almost superimpose. Note that in the SAT phase the total length grows linearly in log *V* for all system sizes as expected but in the UNSAT phase the graphs split. Error bars are smaller than point size.

This makes it suited to be tested on larger graphs. A disadvantage of the greedy method is that it relies heavily on the order in which communications are accommodated (it disregards the information about sender-receiver pairs other than the ones already accommodated). The difference in the performances of the message-passing and greedy algorithm could then be used to assess the relevance of local information usage in such optimization problem. We tested both the standard multi-start and the bounded-length version but we found equal results with the first being slightly faster, in our tests, in terms of execution times. Thus we decided to use the standard MSG in our simulations. First we compared the results in terms of number of accommodated paths *M*_*acc*_ by calculating the ratio *M*_*acc*_/*M*. In Fig 6 we show the behavior of *M*_*acc*_/*M* for each type of random graph and *V* = 10^3^, 〈*k*〉 = 3 using MP, reinforced MP and MSG. Both MP versions perform better than MSG, with the standard MP giving better results. The corresponding results for 〈*k*〉 = 5 are similar (not reported) but the value *M*_*acc*_/*M* < 1 is reached at higher values of *M*/*V* and standard MP and MP with reinforcement give almost always the same solutions. The case 〈*k*〉 = 7 is not reported because, given the high number of edges, the solutions are often trivial (i.e. *M*_*acc*_/*M* = 1), a part from the case of SF graphs where we have instead *M*_*acc*_/*M* < 1 due to the presence of many small degree nodes. We also studied the total path length as a function of *M*/*V* for the solutions, obtained with the different algorithms. We consider the ratio between the total path lengths obtained with greedy and MP for solutions in which the number *M*_*acc*_ of accommodated path is the same. In Fig 7 we can see that MP always outperforms the MSG algorithm for all types of graph under study. The results for the SF graph with 〈*k*〉 = 7 are quite different from the other graphs: both for MP and MSG the ratio departs from 1 at rather small values of *M*/*V*, possibly because the maximum number of accommodated paths is limited by the existence of many small degree nodes that act as bottlenecks, preventing the use of many alternative edge-disjoint routes. The scaling behavior of the fraction 1 − *M*_*acc*_/*M* of unaccommodated communications and the average total path length *L*/*V* of accommodated paths with the system size in the solutions obtained using the MP algorithm is shown in Fig 8 for regular random graphs and ER random graphs. These quantities are plot as functions of the scaling variable $x=\frac{M\log V}{V}$. Note that when paths do not interact, *x* is a measure of the total path length per site, as the average path length is proportional to log *V*. In the top panels, two regimes are visible: for small *x*, all communications can be accommodated, whereas at some value x* the curves for different values of *V* depart from zero. This behavior can be interpreted as a SAT/UNSAT transition, in analogy with the terminology of constraint-satisfaction problems [49]. The collapse of the curves *L*/*V* for different values of *V* is very good in the region in which all paths can be accommodated. On the contrary, in the UNSAT region, the curves for different sizes do not collapse anymore, though the relative difference between them seems to decrease by increasing the system size, and the curves for the largest graphs analyzed (*V* = 8000,10000) are almost superimposed. We argue that *x* is the correct scaling variable in the limit of infinitely large graphs, and the observed mismatch could be due to finite-size effects. The change of slope in the roughly linear behavior of the average total length *L*/*V* is motivated by the fact that in the SAT region, all communications can be accommodated at the cost of taking longer paths with respect to those actually accommodated in the UNSAT region.

## 3 Comparison with other methods

A comparison between the performances of the MP algorithm and those of alternative algorithms proposed in the literature [38, 44] is reported in S1 Table. As benchmark instances we used: two internet-like topologies generated using the BRITE graph generator [65] with parameters set as in [44]; mesh graphs of sizes 15x15 and 25x25, Steiner and planar graphs as reported in [38]. For each of these graphs we used the same set of sender-receiver pairs of size *M* = 0.10*V*,0.25*V*,0.40*V* used in [38]. For each of these instances we ran the MP, MP with reinforcement and MSG algorithms 20 times and collected the average, minimum and maximum number of accommodated paths *M*_*acc*_ along with the average computational time in seconds. All results are reported in S1 Table.

### 3.1 Other optimization methods

A part from the multi-start greedy, we used as comparison two more structured algorithms. The first one is an Ant Colony Optimization metaheuristic [44]. This method builds an EDP solution incrementally from partial solutions provided by a set of *M* ants. Each ant generates a path for a given communication making probabilistic decisions during the construction steps. These are made by processing local information modeled as *pheromone* information provided by other ants. The advantage of this method is to divide the EDP in subproblems and to use local information. The drawback is that it relies on several parameters that need to be carefully tuned in order to have a sensitive solution. Moreover the computational time increases considerably with the system size. The second algorithm is a Montecarlo-based Local Search [38], that uses as main Montecarlo step a path rewiring based on rooted spanning trees. Unfortunately the running time grows rapidly with the system size, making it computationally expensive when used on large graphs. Results are reported in S1 Table. Finally, we performed simulation using the multi-start greedy heuristic described above.

### 3.2 Results

In S1 Table we report the performance comparison in terms of *M*_*acc*_ between the two versions of MP (with and without reinforcement) and the other 3 types of algorithms. The message-passing always performs equal or better than the other methods. The best relative performances are given for meshes and planar graphs, despite the fact that the approximation behind MP is known to be inaccurate in graphs with many short cycles. What we find instead is that, even though the standard MP converges only in a few of these instances on meshes, the version with reinforcement always finds a solution that is no worse than the one of the other algorithms. The largest performance gap is seen on instances with larger set of commodities *M* and generally larger graphs. Performance improvement reaches 27% with respect to LS, overall the best one among the other algorithms tested. Similar considerations can be made in the case of planar graphs. We suspect that this gap may increase with system size, but unfortunately the size of benchmark graphs remains limited to *V* ≤ 500. Moreover these alternative algorithms do not consider path length optimization, thus we cannot compare the performance with respect to this variable. The ACO has been recently tested on several types of graphs (still with *V* ≤ 500) against a Genetic Algorithm (GA) in [42]. It performed better than GA in the case of BRITE graphs 1–6 and 14% worse in the case of 10x10 and 15x15 mesh graphs. The MP algorithm always outperforms ACO and in the case of 15x15 mesh the gap reaches 23.5%. We are not aware of reports on the performance of GA on larger graphs nor results in terms of path length of the solutions.

## 4 Conclusions

Combinatorial optimization problems with edge-disjoint paths find applications in several traffic engineering problems, from VLSI design to routing and access control management in communication networks. In this work we proposed a min-sum message-passing algorithm for the MWEDP problem, in order to find the maximum number of communications *M*_*acc*_ that can be accommodated in a network subject to edge-disjoint constraints and minimizing total path weight at the same time. We devised an efficient method to implement these equations by exploiting a mapping into a minimum weight matching problem on an auxiliary graph. The standard MP algorithm and the version with reinforcement consistently outperform alternative algorithms found in the literature on different types of benchmark graphs in terms of the fraction *M*_*acc*_/*M* of accommodated communications. We found two different behaviors: on some “easy” instances, all algorithm accommodate almost all requests, providing identical results and suggesting that these could be the optimal ones. A second regime comprises non-trivial instances in which *M*_*acc*_/*M* < 1; here the message-passing algorithm always outperforms the other algorithms in terms of the number of accommodated paths, and solutions from different algorithms differ (a fact that could be interpreted as a symptom of “hardness”). Note that in the region with *M*_*acc*_/*M* = 1, the difficulty can clearly only increase with *M*. However, as soon as *M*_*acc*_/*M* < 1, the problem of accommodating those paths that *can* be accommodated could in principle become easier with increasing *M*. Unfortunately, we have no indication one way or the other as true optima are unknown. In particular we obtained better results in the case of meshes and planar graphs, even though these topologies are not locally tree-like as required by the cavity method. In these cases, we could always ensure convergence of the MP equations by exploiting a reinforcement technique. The quality of solutions improves with decreasing the reinforcement parameter, such that we could always find better solutions than those obtained using the other algorithms under study. Unfortunately, for the heuristic algorithms employed on the benchmarks we could not access other relevant metrics such as the average total path length, as it was not considered before in the literature [38, 44]. Nonetheless we could directly compute such quantity for a multi-start greedy heuristic in several graphs, finding that MP always gives a lower average path length for solutions with the same fraction of accommodated communications.

In conclusion, combining the good performance results, in terms of traffic and path length, with the polynomial time implementation, the use of the MP algorithm opens new perspectives in the solution of relevant routing problems over communication networks such as the RWA in optical networks. In particular, it would be interesting to apply the MP algorithm in the iterative construction of RWA solutions over communication networks with finite link capacity, as it has been done for other types of EDP algorithms.

One possible direct generalization is the one of considering an asymmetric weight matrix, i.e. where *w*_*ij*_ is not necessarily equal to *w*_*ji*_. Note that this is different from the directed or bidirected EDP problem, in which for a given couple (*ij*) two paths going in opposite directions can coexist. A generalization to the directed case would be certainly non-trivial, as the problem is NP-Hard even on trees [23].

## Supporting Information

S1 Table Benchmark results. We report the characteristics of the benchmarks and the performance comparison between MP and the other algorithms in terms of the average, the minimum and the max number of accommodated paths over 20 runs of a given set of commodity instance on these networks.
(PDF)

S1 Appendix Convergence criterion. We describe the criterion we used to establish algorithmic convergence in the numerical implementation of the MP equations.
(PDF)

## References

[1] Tanenbaum AS. Computer Networks 4th Edition. Prentice Hall; 2003.

[2] Ash GR. Traffic Engineering and QoS Optimization of Integrated Voice & Data Networks. Morgan Kaufmann; 2006.

[3] Masip-Bruin Xea. Research challenges in QoS routing. Computer communications. 2006;29(5):563–581. 10.1016/j.comcom.2005.06.008

[4] Robertson N, Seymour PD. Graph minors. XIII. The disjoint paths problem. Journal of combinatorial theory, Series B. 1995;63(1):65–110. 10.1006/jctb.1995.1006

[5] Gerez SH. Algorithms for VLSI design automation. vol. 8 Wiley Chichester, England; 1999.

[6] Chan WT, Chin FY, Ting HF. Escaping a grid by edge-disjoint paths. Algorithmica. 2003;36(4):343–359. 10.1007/s00453-003-1023-8

[7] Awerbuch B, Gawlick R, Leighton T, Rabani Y. On-line admission control and circuit routing for high performance computing and communication. In: Foundations of Computer Science, 1994 Proceedings., 35th Annual Symposium on. IEEE; 1994; 412–423.

[8] Gerla M, Tsai JTC. Multicluster, mobile, multimedia radio network. Wireless networks. 1995;1(3):255–265. 10.1007/BF01200845

[9] Akkaya K, Younis M. A survey on routing protocols for wireless sensor networks. Ad hoc networks. 2005;3(3):325–349. 10.1016/j.adhoc.2003.09.010

[10] Shin KG, Ramanathan P. Real-time computing: A new discipline of computer science and engineering. Proceedings of the IEEE. 1994;82(1):6–24. 10.1109/5.259423

[11] Mahapatra A, Anand K, Agrawal DP. QoS and energy aware routing for real-time traffic in wireless sensor networks. Computer Communications. 2006;29(4):437–445. 10.1016/j.comcom.2004.12.028

[12] Salama HF, Reeves DS, Viniotis Y. Evaluation of multicast routing algorithms for real-time communication on high-speed networks. Selected Areas in Communications, IEEE Journal on. 1997;15(3):332–345. 10.1109/49.564132

[13] Sumpter Z, Burson L, Tang B, Chen X; IEEE. Maximizing Number of Satisfiable Routing Requests in Static Ad Hoc Networks. IEEE GLOBECOM 2013 Conference Proceedings. 2013.

[14] Srinivas A, Modiano E. Minimum energy disjoint path routing in wireless ad-hoc networks. In: Proceedings of the 9th annual international conference on Mobile computing and networking. ACM; 2003; 122–133.

[15] Jain S, Das SR. Exploiting path diversity in the link layer in wireless ad hoc networks. Ad Hoc Networks. 2008;6(5):805–825. 10.1016/j.adhoc.2007.07.002

[16] Li X, Cuthbert L. Node-disjointness-based Multipath Routing for Mobile Ad Hoc Networks. In: Proceedings of the 1st ACM International Workshop on Performance Evaluation of Wireless Ad Hoc, Sensor, and Ubiquitous Networks. PE-WASUN’04. ACM; 2004; 23–29.

[17] Chatterjee BC, Sarma N, Sahu PP, et al Review and performance analysis on routing and wavelength assignment approaches for optical networks. IETE Technical Review. 2013;30(1):12.

[18] Manohar P, Manjunath D, Shevgaonkar R. Routing and wavelength assignment in optical networks from edge disjoint path algorithms. Comm Lett, IEEE. 2002;6(5):211–213. 10.1109/4234.1001667

[19] Hsu CC, Cho HJ, Fang SC. Routing and Wavelength Assignment in Optical Networks from Maximum Edge-Disjoint Paths In: Genetic and Evolutionary Computing. Springer; 2014; 95–103.

[20] Karp RM. Reducibility among combinatorial problems. Springer; 1972.

[21] Garey MR, Johnson DS. Computers and Intractability: A Guide to the Theory of NP-Completeness. Series of books in the mathematical sciences. W. H. Freeman; 1979.

[22] Vygen J. Disjoint paths. Citeseer; 1994. Available: http://citeseerx.ist.psu.edu/viewdoc/download?doi=10.1.1.53.268&rep=rep1&type=pdf

[23] Erlebach T. Approximation algorithms for edge-disjoint paths and unsplittable flow In: Efficient Approximation and Online Algorithms. Springer; 2006; 97–134.

[24] Chuzhoy J, Khanna S. New hardness results for undirected edge disjoint paths. 2005; Available: http://ttic.uchicago.edu/˜cjulia/papers/edpc.pdf

[25] Chekuri C, Khanna S, Shepherd FB. An $O(\sqrt{n})$ approximation and integrality gap for disjoint paths and unsplittable flow. Theory of computing. 2006;2(7):137–146. 10.4086/toc.2006.v002a007

[26] Garg N, Vazirani VV, Yannakakis M. Primal-dual approximation algorithms for integral flow and multicut in trees. Algorithmica. 1997;18(1): 3–20. 10.1007/BF02523685

[27] Awerbuch B, Gawlick R, Leighton T, Rabani Y. On-line admission control and circuit routing for high performance computing and communication. In: Foundations of Computer Science, 1994 Proceedings., 35th Annual Symposium on. IEEE; 1994; 412–423.

[28] Aumann Y, Rabani Y. Improved bounds for all optical routing. In: Proceedings of the sixth annual ACM-SIAM symposium on Discrete algorithms. Society for Industrial and Applied Mathematics; 1995; 567–576.

[29] Kleinberg J. An approximation algorithm for the disjoint paths problem in even-degree planar graphs. In: Foundations of Computer Science, 2005. FOCS 2005. 46th Annual IEEE Symposium on. IEEE; 2005; 627–636.

[30] Kleinberg J, Tardos E. Disjoint paths in densely embedded graphs. In:, 36th Annual Symposium on Foundations of Computer Science, 1995. Proceedings; 1995; 52–61.

[31] Chen C, Banerjee S. A new model for optimal routing and wavelength assignment in wavelength division multiplexed optical networks. In: INFOCOM’96. Fifteenth Annual Joint Conference of the IEEE Computer Societies. Networking the Next Generation. Proc. IEEE. vol. 1. IEEE; 1996; 164–171.

[32] Skorin-Kapov N. Routing and wavelength assignment in optical networks using bin packing based algorithms. Eur J Op Res. 2007;177(2):1167–1179. 10.1016/j.ejor.2006.01.003

[33] Banerjee D, Mukherjee B. A practical approach for routing and wavelength assignment in large wavelength-routed optical networks. Sel Ar in Comm, IEEE. 1996;14(5):903–908. 10.1109/49.510913

[34] Kolliopoulos SG, Stein C. Approximating disjoint-path problems using packing integer programs. Mathematical Programming. 2004;99(1):63–87. 10.1007/s10107-002-0370-6

[35] Ozdaglar AE, Bertsekas DP. Routing and wavelength assignment in optical networks. IEEE/ACM Transactions on Networking (TON). 2003;11(2):259–272. 10.1109/TNET.2003.810321

[36] Baveja A, Srinivasan A. Approximation algorithms for disjoint paths and related routing and packing problems. Mathematics of Operations Research. 2000;25(2):255–280. 10.1287/moor.25.2.255.12228

[37] Belgacem L, Charon I, Hudry O. A post-optimization method for the routing and wavelength assignment problem applied to scheduled lightpath demands. Eur J Op Res. 2014;232(2):298–306. 10.1016/j.ejor.2013.06.050

[38] Pham QD, Deville Y, Van Hentenryck P. LS (Graph): a constraint-based local search for constraint optimization on trees and paths. Constraints. 2012;17(4):357–408. 10.1007/s10601-012-9124-0

[39] Noronha TF, Resende MG, Ribeiro CC. A biased random-key genetic algorithm for routing and wavelength assignment. Journal of Global Optimization. 2011;50(3):503–518. 10.1007/s10898-010-9608-7

[40] Gen M, Cheng R, Lin L. Network models and optimization: Multiobjective genetic algorithm approach. Springer; 2008.

[41] Storn R, Price K. Differential evolution–a simple and efficient heuristic for global optimization over continuous spaces. J glob opt. 1997;11(4):341–359. 10.1023/A:1008202821328

[42] Hsu CC, Cho HJ. A Genetic Algorithm for the Maximum Edge-disjoint Paths Problem. Neurocomputing. 2014.

[43] Hassan A, Phillips C, Pitts J. Dynamic routing and wavelength assignment using hybrid particle swarm optimization for wdm networks. In: EPSRC PostGraduate Network Symposium (PGNet); 2007.

[44] Blesa M, Blum C. Ant colony optimization for the maximum edge-disjoint paths problem In: App. Ev. Comp. Springer; 2004; 160–169.

[45] Mézard M, Parisi G. The cavity method at zero temperature. J Stat Phys. 2003;111(1–2):1–34.

[46] Mézard M, Parisi G, Virasoro MA. Spin glass theory and beyond. vol. 9. World scientific Singapore; 1987.

[47] Mézard M, Montanari A. Information, physics, and computation. Oxford University Press; 2009.

[48] Mézard M, Parisi G. The Bethe lattice spin glass revisited. Eur J Phys B. 2001;20(2):217–233. 10.1007/PL00011099

[49] Mézard M, Parisi G, Zecchina R. Analytic and algorithmic solution of random satisfiability problems. Science. 2002;297(5582):812–815. 10.1126/science.1073287 12089451

[50] Yeung CH, Saad D. Competition for shortest paths on sparse graphs. Phys Rev Lett. 2012;108(20):208701 10.1103/PhysRevLett.108.208701 23003195

[51] Yeung CH, Saad D, Wong KM. From the physics of interacting polymers to optimizing routes on the London Underground. Proc Nat Ac Sci. 2013;110(34):13717–13722. 10.1073/pnas.1301111110 PMC3752220 23898198

[52] Bayati M, Borgs C, Braunstein A, Chayes J, Ramezanpour A, Zecchina R. Statistical mechanics of steiner trees. Physical review letters. 2008;101(3):037208 10.1103/PhysRevLett.101.037208 18764290

[53] De Bacco C, Franz S, Saad D, Yeung CH. Shortest node-disjoint paths on random graphs. Journal of Statistical Mechanics: Theory and Experiment. 2014;2014(7):P07009 Available: http://iopscience.iop.org/1742-5468/2014/7/P07009 10.1088/1742-5468/2014/07/P07009

[54] Lovász L, Plummer D. Matching Theory. AMS Chelsea Publishing Series. American Mathematical Soc.; 2009.

[55] Bayati M, Shah D, Sharma M. Max-Product for Maximum Weight Matching: Convergence, Correctness, and LP Duality. IEEE Transactions on Information Theory. 2008 3;54(3):1241–1251. Available: http://ieeexplore.ieee.org/lpdocs/epic03/wrapper.htm?arnumber=4455730 10.1109/TIT.2007.915695

[56] Bayati M, Braunstein A, Zecchina R. A rigorous analysis of the cavity equations for the minimum spanning tree. Journal of Mathematical Physics. 2008;49(12):125206 Available: http://link.aip.org/link/?JMP/49/125206/1 10.1063/1.2982805

[57] Gamarnik D, Shah D, Wei Y. Belief Propagation for Min-Cost Network Flow: Convergence & Correctness. Society for Industrial and Applied Mathematics; 2010. National Science Foundation (U.S.) (Project CMMI-0726733). Available: http://dspace.mit.edu/handle/1721.1/73521

[58] Erlebach T, Jansen K. The maximum edge-disjoint paths problem in bidirected trees. SIAM Journal on Discrete Mathematics. 2001;14(3):326–355. 10.1137/S0895480199361259

[59] Galil Z, Micali S, Gabow H. An *O*(*EV*∖*logV*) Algorithm for Finding a Maximal Weighted Matching in General Graphs. SIAM Journal on Computing. 1986;15(1):120–130. 10.1137/0215009

[60] Braunstein A, Zecchina R. Learning by message passing in networks of discrete synapses. Physical review letters. 2006;96(3):030201 10.1103/PhysRevLett.96.030201 16486667

[61] Altarelli F, Braunstein A, Realpe-Gomez J, Zecchina R. Statistical mechanics of budget-constrained auctions. Journal of Statistical Mechanics: Theory and Experiment. 2009;2009(07):P07002 10.1088/1742-5468/2009/07/P07002

[62] Erdős P, Rényi A. On the evolution of random graphs. Publications of the Mathematical Institute of the Hungarian Academy of Sciences. 1960;5:17–61.

[63] Newman ME, Strogatz SH, Watts DJ. Random graphs with arbitrary degree distributions and their applications. Physical Review E. 2001;64(2):026118 10.1103/PhysRevE.64.026118 11497662

[64] Kleinberg JM. Approximation algorithms for disjoint paths problems. Citeseer; 1996.

[65] Medina A, Lakhina A, Matta I, Byers J. BRITE: An approach to universal topology generation. In: Modeling, Analysis and Simulation of Computer and Telecommunication Systems, 2001. Proceedings. Ninth International Symposium on. IEEE; 2001. p. 346–353. Available: http://www.cs.bu.edu/brite/

