# Supplementary material for: The Edge-Disjoint Path Problem on Random Graphs by Message-Passing
Source: PLoS One. 2015 Dec 28;10(12):e0145222. doi: 10.1371/journal.pone.0145222 (PMC4699204; doi:10.1371/journal.pone.0145222)
Supplement: S1 Table — We report the characteristics of the benchmarks and the performance comparison between MP and the other algorithms in terms of the average, the minimum and the max number of accommodated paths over 20 runs of a given set of commodity instance on these networks. (PDF) [file pone.0145222.s001.pdf]

S1 Table. Benchmark results.

Instance				MP			MP rein = 0.002			MSG (greedy)			ACO			LS			MP gain vs.		
Name	$\|V\|$	$\|E\|$	$\langle k \rangle$	$\langle M \rangle$	M_{min}	M_{max}	$\langle M \rangle$	M_{min}	M_{max}	$\langle M \rangle$	M_{min}	M_{max}	$\langle M \rangle$	M_{min}	M_{max}	$\langle M \rangle$	M_{min}	M_{max}	MSG	ACO	LS
blrand10_1	500	1020	4.08	16.00	16	**16**	16.00	16	**16**	13.65	13	15	14.80	14	**16**	16.00	16	**16**	6.67	0.00	0.00
blrand25_1	500	1020	4.08	32.00	32	**32**	32.00	32	**32**	27.75	26	30	31.85	31	**32**	32.00	32	**32**	6.67	0.00	0.00
blrand40_1	500	1020	4.08	38.00	38	**38**	38.00	38	**38**	33.10	32	35	37.85	37	**38**	37.90	37	**38**	8.57	0.00	0.00
blrand10_2	500	1020	4.08	26.00	26	**26**	25.65	25	**26**	23.85	23	25	25.25	25	**26**	26.00	26	**26**	4.00	0.00	0.00
blrand25_2	500	1020	4.08	35.00	35	**35**	35.00	35	**35**	30.75	29	33	34.75	34	**35**	34.95	34	**35**	6.06	0.00	0.00
blrand40_2	500	1020	4.08	37.00	37	**37**	37.00	37	**37**	32.45	31	34	36.95	36	**37**	36.95	36	**37**	8.82	0.00	0.00
blsdeg10_1	500	1020	4.08	17.00	17	**17**	16.89	15	**17**	14.65	14	16	15.95	15	16	17.00	17	**17**	6.25	6.25	0.00
blsdeg25_1	500	1020	4.08	36.00	36	**36**	36.00	36	**36**	31.55	30	33	35.80	35	**36**	36.00	36	**36**	9.09	0.00	0.00
blsdeg40_1	500	1020	4.08	34.00	34	**34**	34.00	34	**34**	29.00	28	31	33.65	33	**34**	34.00	34	**34**	9.68	0.00	0.00
blsdeg10_2	500	1020	4.08	20.00	20	**20**	19.85	19	**20**	16.90	16	18	19.20	19	**20**	20.00	20	**20**	11.11	0.00	0.00
blsdeg25_2	500	1020	4.08	34.00	34	**34**	34.00	34	**34**	28.45	27	30	32.95	32	**34**	33.90	33	**34**	13.33	0.00	0.00
blsdeg40_2	500	1020	4.08	37.00	37	**37**	37.00	37	**37**	31.75	30	33	36.50	35	**37**	37.00	37	**37**	12.12	0.00	0.00
mesh15_10_1	225	420	3.73	22.00	22	**22**	22.00	22	**22**	20.60	20	**22**	19.65	19	21	21.55	21	**22**	0.00	4.76	0.00
mesh15_25_1	225	420	3.73	36.00	36	**36**	35.10	35	**36**	28.30	27	30	27.70	26	29	32.00	31	33	20.00	24.14	9.09
mesh15_40_1	225	420	3.73	43.00	43	**43**	42.50	42	**43**	30.10	28	32	35.30	32	38	38.80	37	40	34.38	13.16	7.50
mesh15_10_2	225	420	3.73	-	-	-	19.89	19	**20**	19.75	19	**20**	17.50	17	19	19.45	19	**20**	0.00	5.26	0.00
mesh15_25_2	225	420	3.73	35.00	35	**35**	34.70	33	**35**	29.25	29	30	29.20	28	31	33.05	32	34	16.67	12.90	2.94
mesh15_40_2	225	420	3.73	42.00	42	**42**	41.35	41	**42**	29.80	29	32	34.00	33	36	37.60	36	39	31.25	16.67	7.69
mesh25_10_1	625	1200	3.84	-	-	-	47.25	46	**48**	40.70	40	42	32.85	29	36	41.00	39	43	14.29	33.33	11.63
mesh25_25_1	625	1200	3.84	-	-	-	68.30	67	**69**	48.40	47	51	45.00	42	49	55.55	54	59	35.29	40.82	16.95
mesh25_40_1	625	1200	3.84	-	-	-	88.74	88	**90**	54.35	53	58	57.70	53	61	69.30	67	72	55.17	47.54	25.00
mesh25_10_2	625	1200	3.84	-	-	-	44.33	43	**46**	40.05	38	42	30.10	28	33	37.90	36	40	9.52	39.39	15.00
mesh25_25_2	625	1200	3.84	-	-	-	67.22	65	**70**	48.90	47	52	45.60	44	48	54.70	52	59	34.62	45.83	18.64
mesh25_40_2	625	1200	3.84	-	-	-	88.55	87	**90**	54.05	51	57	57.75	54	61	68.85	66	71	57.89	47.54	26.76
steinb4_10	50	100	4.00	5.00	5	**5**	5.00	5	**5**	5.00	5	**5**	5.00	5	**5**	5.00	5	**5**	0.00	0.00	0.00
steinb4_25	50	100	4.00	12.00	12	**12**	12.00	12	**12**	12.00	12	**12**	12.00	12	**12**	12.00	12	**12**	0.00	0.00	0.00
steinb4_40	50	100	4.00	20.00	20	**20**	20.00	20	**20**	20.00	20	**20**	20.00	20	**20**	19.90	19	**20**	0.00	0.00	0.00
steinb10_10	75	150	4.00	7.00	7	**7**	7.00	7	**7**	7.00	7	**7**	7.00	7	**7**	7.00	7	**7**	0.00	0.00	0.00
steinb10_25	75	150	4.00	18.00	18	**18**	18.00	18	**18**	18.00	18	**18**	17.85	17	**18**	18.00	18	**18**	0.00	0.00	0.00
steinb10_40	75	150	4.00	28.00	28	28	27.65	27	**29**	25.10	24	27	24.35	23	26	27.30	27	28	7.41	11.54	3.57
steinb16_10	100	200	4.00	10.00	10	**10**	10.00	10	**10**	10.00	10	**10**	10.00	10	**10**	10.00	10	**10**	0.00	0.00	0.00
steinb16_25	100	200	4.00	25.00	25	**25**	25.00	25	**25**	25.00	25	**25**	24.35	24	**25**	25.00	25	**25**	0.00	0.00	0.00
steinb16_40	100	200	4.00	36.12	36	**37**	36.00	36	36	33.20	32	34	32.45	32	34	35.95	35	**37**	8.82	8.82	0.00
steinc6_10	500	1000	4.00	50.00	50	**50**	50.00	50	**50**	50.00	50	**50**	49.10	47	**50**	50.00	50	**50**	0.00	0.00	0.00
steinc6_25	500	1000	4.00	125.00	125	**125**	122.55	121	124	107.50	106	110	89.90	85	94	104.95	102	108	13.64	32.98	15.74
stienc6_40	500	1000	4.00	145.84	144	**147**	140.40	139	142	114.10	112	117	109.80	106	117	121.40	119	125	25.64	25.64	17.60
steincc11_10	500	2500	10.00	50.00	50	**50**	50.00	50	**50**	50.00	50	**50**	50.00	50	**50**	50.00	50	**50**	0.00	0.00	0.00
steinc11_25	500	2500	10.00	125.00	125	**125**	125.00	125	**125**	125.00	125	**125**	123.30	122	**125**	125.00	125	**125**	0.00	0.00	0.00
steinc11_40	500	2500	10.00	200.00	200	**200**	200.00	200	**200**	200.00	200	**200**	194.25	190	198	200.00	200	**200**	0.00	1.01	0.00
steinc16_10	500	12500	50.00	50.00	50	**50**	50.00	50	**50**	50.00	50	**50**	50.00	50	**50**	50.00	50	**50**	0.00	0.00	0.00
steinc16_25	500	12500	50.00	-	-	-	125	125	**125**	125.00	125	**125**	125.00	125	**125**	125.00	125	**125**	0.00	0.00	0.00
steinc16_40	500	12500	50.00	-	-	-	200	200	**200**	200.00	200	**200**	200.00	200	**200**	200.00	200	**200**	0.00	0.00	0.00
plan50_10	50	135	5.40	5.00	5	**5**	5.00	5	**5**	5.00	5	**5**	5.00	5	**5**	5.00	5	**5**	0.00	0.00	0.00
plan50_25	50	135	5.40	12.00	12	**12**	12.00	12	**12**	12.00	12	**12**	12.00	12	**12**	12.00	12	**12**	0.00	0.00	0.00
plan50_40	50	135	5.40	20.00	20	**20**	20.00	20	**20**	20.00	20	**20**	20.00	20	**20**	19.90	19	**20**	0.00	0.00	0.00
plan100_10	100	285	5.70	10.00	10	**10**	10.00	10	**10**	10.00	10	**10**	10.00	10	**10**	10.00	10	**10**	0.00	0.00	0.00
plan100_25	100	285	5.70	25.00	25	**25**	25.00	25	**25**	25.00	25	**25**	25.00	25	**25**	25.00	25	**25**	0.00	0.00	0.00
plan100_40	100	285	5.70	37.00	37	37	37.05	37	**38**	35.80	35	37	34.00	33	36	36.00	35	37	2.70	5.56	2.70
plan200_10	200	583	5.83	20.00	20	**20**	20.00	20	**20**	20.00	20	**20**	20.00	20	**20**	20.00	20	**20**	0.00	0.00	0.00
pan200_25	200	583	5.83	-	-	-	48.95	48	**50**	46.50	46	48	41.80	39	43	45.95	45	48	4.17	16.28	4.17
plan200_40	200	583	5.83	-	-	-	60.65	58	**62**	52.95	52	56	49.35	47	51	55.70	54	58	10.71	21.57	6.90
plan500_10	500	1477	5.91	50.00	50	**50**	50.00	50	**50**	50.00	50	**50**	44.95	42	47	50.00	50	**50**	0.00	6.38	0.00
plan500_25	500	1477	5.91	-	-	-	92.29	90	**94**	78.15	76	80	60.95	57	65	78.20	77	80	17.50	44.62	17.50
plan500_40	500	1477	5.91	-	-	-	122.31	119	**124**	92.60	90	95	82.85	78	86	100.15	97	102	30.53	44.19	21.57

Table 1: Message-passing and multi-start greedy performances. Columns 1-4 give the characteristics of the benchmark. For each algorithm, columns 1-3 represent the average, the minimum and the max number of accommodated paths over 20 runs of a given set of commodity instance respectively. ACO and LS performances are reported in [1, 2]. Performance comparison between MP and the other algorithms is given in the three last columns, representing the performance ratio $100 \cdot (M_{acc}^{BP}/M_{acc}^{alg} - 1)$ where alg indicates the algorithm used (MSG, ACO and LS respectively). We use as M_{acc}^{MP} the best one between MP with and without reinforcement.

References

[1] Blesa M, Blum C. Ant colony optimization for the maximum edge-disjoint paths problem. In: App. Ev. Comp. Springer; 2004. p. 160–169.

[2] Pham QD, Deville Y, Van Hentenryck P. LS (Graph): a constraint-based local search for constraint optimization on trees and paths. Constraints. 2012;17(4):357–408.
