# Supplementary material for: The Edge-Disjoint Path Problem on Random Graphs by Message-Passing
Source: PLoS One. 2015 Dec 28;10(12):e0145222. doi: 10.1371/journal.pone.0145222 (PMC4699204; doi:10.1371/journal.pone.0145222)
Supplement: S1 Appendix — We describe the criterion we used to establish algorithmic convergence in the numerical implementation of the MP equations. (PDF) [file pone.0145222.s002.pdf]

S1 Appendix. Convergence criterion.

Given a decision variable d^t to be calculated at each iteration update step t, an integer variable n and a time step T_{max} we have convergence if, for n consecutive iteration steps, d^t does not change, and we fix a maximum iteration time T_{max} to update MP equations. Formally this writes:

$$\exists\, t_0 \in [1, T_{max} - n] \quad s.t. \quad d^{t_0+i} = d^{t_0} \quad \forall i = 1, \dots, n \tag{1}$$

In our simulations we defined the decision variable as the total difference of the optimal currents (calculated edge by edge) between two consecutive iteration steps :

$$d^t = \sum_{(ij)\in E} [1 - \delta_{\mu_{ij}^t, \mu_{ij}^{t-1}}] \tag{2}$$

where $\mu_{ij}^t = |\min_{\mu=-M,\dots,M} \{E_{ij}(\mu) + E_{ji}(-\mu) - c_{ij}(\mu)\}\,|$ and convergence is reached when $d^t = 0$ for n consecutive time steps.
